# Integrating spatial and single-cell transcriptomics reveals tumor heterogeneity and intercellular networks in colorectal cancer

**DOI:** 10.1038/s41419-024-06598-6

**Published:** 2024-05-10

**Authors:** Jing Xiao, Xinyang Yu, Fanlin Meng, Yuncong Zhang, Wenbin Zhou, Yonghong Ren, Jingxia Li, Yimin Sun, Hongwei Sun, Guokai Chen, Ke He, Ligong Lu

**Affiliations:** 1 Guangdong Provincial Key Laboratory of Tumor Interventional Diagnosis and Treatment, Zhuhai People’s Hospital, (Zhuhai Clinical Medical College of Jinan University), Jinan University, Zhuhai, Guangdong China; 2 Centre of Reproduction, Development and Aging, Faculty of Health Sciences, University of Macau, Macau SAR, China; 3 CapitalBio Technology Corporation, Beijing, China; 4 Zhuhai UM Science & Technology Research Institute, Zhuhai, Guangdong China; 5 Minimally Invasive Tumor Therapies Center, Guangdong Second Provincial General Hospital, Guangzhou, Guangdong China; 6 Guangzhou First People’s Hospital, the Second Affiliated Hospital, School of Medicine, South China University of Technology, Guangzhou, Guangdong China

**Keywords:** Cancer microenvironment, Cell adhesion

## Abstract

Single cell RNA sequencing (scRNA-seq), a powerful tool for studying the tumor microenvironment (TME), does not preserve/provide spatial information on tissue morphology and cellular interactions. To understand the crosstalk between diverse cellular components in proximity in the TME, we performed scRNA-seq coupled with spatial transcriptomic (ST) assay to profile 41,700 cells from three colorectal cancer (CRC) tumor-normal-blood pairs. Standalone scRNA-seq analyses revealed eight major cell populations, including B cells, T cells, Monocytes, NK cells, Epithelial cells, Fibroblasts, Mast cells, Endothelial cells. After the identification of malignant cells from epithelial cells, we observed seven subtypes of malignant cells that reflect heterogeneous status in tumor, including tumor_CAV1, tumor_ATF3_JUN | FOS, tumor_ZEB2, tumor_VIM, tumor_WSB1, tumor_LXN, and tumor_PGM1. By transferring the cellular annotations obtained by scRNA-seq to ST spots, we annotated four regions in a cryosection from CRC patients, including tumor, stroma, immune infiltration, and colon epithelium regions. Furthermore, we observed intensive intercellular interactions between stroma and tumor regions which were extremely proximal in the cryosection. In particular, one pair of ligands and receptors (C5AR1 and RPS19) was inferred to play key roles in the crosstalk of stroma and tumor regions. For the tumor region, a typical feature of *TMSB4X*-high expression was identified, which could be a potential marker of CRC. The stroma region was found to be characterized by *VIM*-high expression, suggesting it fostered a stromal niche in the TME. Collectively, single cell and spatial analysis in our study reveal the tumor heterogeneity and molecular interactions in CRC TME, which provides insights into the mechanisms underlying CRC progression and may contribute to the development of anticancer therapies targeting on non-tumor components, such as the extracellular matrix (ECM) in CRC. The typical genes we identified may facilitate to new molecular subtypes of CRC.

## Introduction

Colorectal cancer (CRC) is a molecularly heterogeneous disease [1, 2]. The heterogeneity of cell types involved in CRC carcinogenesis makes it difficult to elucidate cell lineages using traditional developmental biology techniques such as bulk transcriptomics methods [3]. Through single-cell transcriptomics technology, it is now possible to deconstruct a tumor into its diverse cell subpopulations and thus gain a better understanding of the underlying biology like subtyping [4–6]. However, spatial or anatomical information inherent in the tissue architecture is lost using single-cell transcriptomic technology only.

Spatial transcriptomics (ST) is an emerging technology that adds spatial dimensionality and tissue morphology information to the single-cell transcriptomics data of cells in an undissociated tissue, thus helping to preserve precise spatial or anatomical information. Overcoming the throughput limitation of in situ hybridization (ISH) methods, ST allows for unbiased mapping of transcripts in individual tissue sections with spatial resolution by using spatially barcoded oligo-deoxythymidine microarrays [7]. As a high-throughput spatially resolved transcriptomic tool, ST has been used to study architecturally complex tissues or diseases including melanoma [8], prostate cancer [9], cardiac sarcoidosis [10], non-small cell lung [11], human and other species’ cortex [12, 13], as well as their spatiotemporal characterizations [14–16].

Extensive multimodal studies have unraveled molecular landscape of diverse diseases [17]. Combining these two complementary and powerful technologies has been confirmed to be scalable to study architecturally complex tissues and to provide meaningful biological insight across a range of pathologies, such as melanoma [18], bone marrow [19], prostate cancer [20], pancreatic ductal adenocarcinomas [21], myocardial infarction [22], lung fibroblasts [23], spinal cord [24] and plants like rice root [25].

The tumor microenvironment (TME) comprises various cell types (immune cells, fibroblasts, endothelial cells, etc.) and extracellular components (growth factors, cytokines, extracellular matrix, hormones, etc.) that surround cancerous/tumor cells [26]. Since many currently used anticancer therapies target non-tumor components, such as the extracellular matrix (ECM) [27], immune system and vascular system [28], understanding cellular components and how their dynamic interactions to shape the tumor landscape are particularly important.

In this study, we aim to provide a comprehensive global view of tumor heterogeneity and intercellular interaction networks of CRC using single-cell transcriptional profiles coupled with spatial transcriptional profiles. By analyzing the single-cell and spatial transcriptional profiles of 41,700 cells from 3 treatment-naïve patients with CRC, we generated a molecular map of all major CRC populations based on single-cell RNA sequencing (scRNA-seq). The malignant cells in epithelial cells were identified and categorized into seven subclasses (tumor_CAV1, tumor_ATF3_JUN | FOS, tumor_ZEB2, tumor_VIM, tumor_WSB1, tumor_LXN, tumor_PGM1), which may help to the molecular subtyping of colorectal cancers. In addition, we used spatially resolved transcriptomics in combination with computational tools to attribute cell types to different CRC niches. Annotated tumor regions based on the cryosection sections represented high *TMSB4X* expression, and suggested a typical marker of tumorgenesis. The stroma region was characterized by *VIM* gene, which was also used as a typical feature of one subtype of malignant cells in CRC scRNA-seq. Furthermore, we inferred the important interaction between tumor and stromal regions mediated by gene pair of C5AR1 and RPS19, which played roles of ligand and receptor, respectively.

## Results

### Landscape view of cell composition in tumors, adjacent tissues and peripheral blood in patients with CRC

**Fig. 1:**
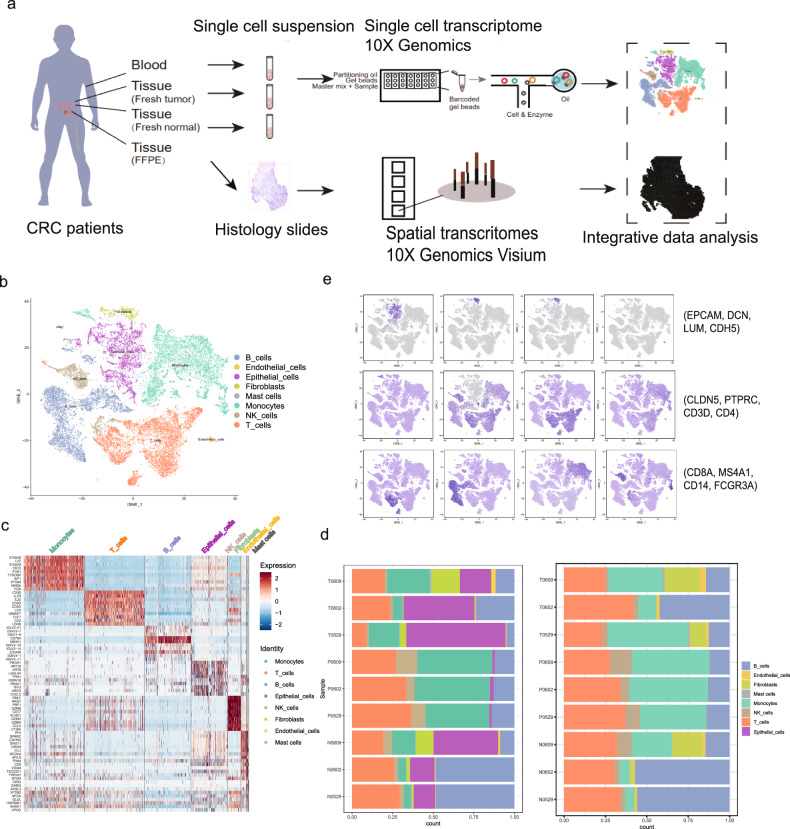
Cell type identification in human CRC by 10X Genomics scRNA-seq. **a** Workflow of sample collection and single-cell transcriptome analysis from Chinese patients with CRC. **b** t-distributed stochastic neighbor embedding (t-SNE) plot of 35,666 high-quality cells from CRC patients (the CRC scRNA-seq dataset), grouped into eight major cell types (left panel, top). Proportions of the global cell types in tumor tissues, adjacent tissues and blood on average (left panel, bottom). The normalized expression of marker genes for each cell type (right panel) (**c**). Gene expression heatmap analyzed by 10X Genomics scRNA-seq. **d** Proportions of the global cell types in individual samples with CRC.

To shed light on the complexity of the TME in CRC, we performed scRNA-seq along with spatial trancriptome sequencing on viable cells derived from matched tumor and adjacent tissues, as well as peripheral blood mononuclear cells (PBMCs) of 3 patients with CRC (Fig. 1a, Supplementary Table S1). On average, we obtained more than 150 G sequencing reads for each sample, with a median sequencing saturation of 91.40% (87.0%–95.5%). A total of 41,700 cells were identified in 9 samples derived from 3 patients (including 10347, 13241 and 18112 in tumor tissues, adjacent tissues and peripheral blood, respectively; Supplementary Table S2). We obtained approximately 1000 genes and 2500 unique molecular identifiers (UMIs) for each cell, indicating sufficient coverage and transcript representations. After quality control filters (few detected features in cells and few expressed cells associated with detected features), we acquired 35,666 high-quality cells for further analysis.

To define cell clusters with similar expression profiles, we performed dimensionality reduction of t-distributed stochastic neighbor embedding (tSNE) implemented in the Seurat package. Each cluster was further identified as a specific cell subpopulation on the basis of the expression of the most variable genes and the canonical markers, including those in epithelial cells (with gene markers: EPCAM, KRT5, PHGR1, LGALS4, and TFF3), T cells (CD4 + T cells: PTPRC, CD3D, and CD4; CD8 + T cells: PTPRC, CD3D, and CD8A), B cells (CD19 and MS4A1), monocytes (CD14, ITGAX for CD11C), natural killer (NK) cells (FCGR3A and NCAM1), endothelial cells (CDH5, PLVAP, CLDN5, VWF), fibroblasts (LUM, DCN, COL1A1), and mast cells (KIT, CPA3, MS4A2, and TPSAB1) (Fig. 1b). In addition to these well-known markers, we also analyzed cluster-specific genes via differential gene expression analysis (Supplementary Table S3). These cluster-specific marker genes included FBLN1 for fibroblasts, as well as MT1A and PLN for smooth muscle cells (Fig. 1c, Fig. S1e). In total, eight cell types in CRC were identified based on canonical markers and cluster-specific genes: epithelial cells, fibroblasts, endothelial cells, monocytes, T cells, NK cells, B cells, and mast cells. The heterogeneous compositions of the TME in CRC across tumor tissues, normal tissues and peripheral blood are consistent with a recent single-cell transcriptome study of CRC [29].

To characterize different cell compositions in tumor tissues, normal tissues and peripheral blood in CRC, the proportions of each cell type were investigated. An overall increase in myeloid cell populations and decrease in B cell populations were observed in tumor tissues compared to normal tissues (Fig. 1b, bottom; Fig. S1d), suggesting a redirected immune response in CRC patients. In details, we observed that the proportion of monocytes was increased with approximately 2.5-fold, whereas that of NK cells and B cells was decreased (about 0.3–0.4 times) in tumors compared to normal tissues, suggesting a myeloid immunosuppression in the CRC TME (Fig. S1a,b, Supplementary Table S4). To further explore the distinct cell compositions in the TME across individuals, more detailed proportions were assessed (Fig. 1d). These results showed, for example, that in patient T0602, the proportion of epithelial cells decreased in contrast with patient T0529 and increased compared to that in patient T0609 (Fig. 1d, left; Fig. S1 c1; Supplementary Table S5). Since the transition from normal epithelium to intraepithelial neoplasia were found to be associated with CRC patient survival [30, 31], the difference in epithelial cells across individuals may be important for survival and worthy of further investigation. Considering that the cellular proportion determined by scRNA-seq may be biased toward an underrepresentation of malignant cells derived from epithelial cells [32], we also explored the proportions of immune and stromal cells account for all cells except epithelial cells which includes tumor cells like previous study [29]. The results showed that myeloid cell-driven immune response in patient T0529 was stronger than that in the other two patients (Fig. 1d, right; Fig. S1 c2).

### Epithelial cells represents multilineages including a lineage of malignant cells

**Fig. 2:**
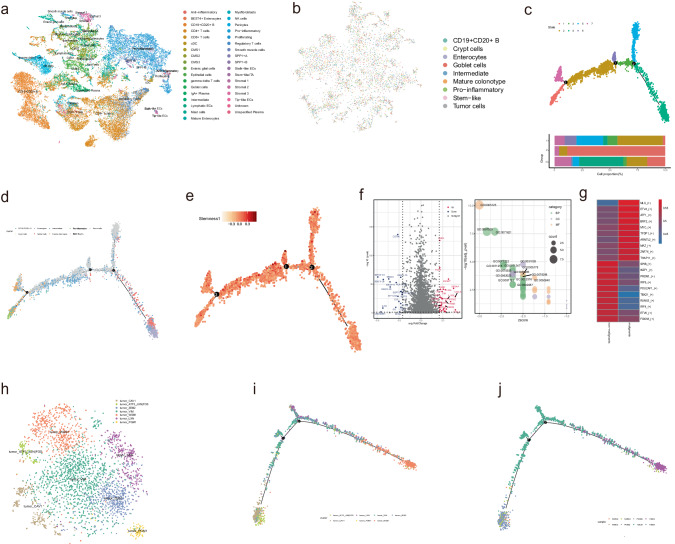
Transcriptome signatures and heterogeneity in normal and tumor epithelial cells. **a** t-SNE plot of the CRC scRNA-seq dataset color-coded by colorectal subtypes. **b** t-SNE plot of all 5887 epithelial cells (tumor/malignant cells are included) of the CRC scRNA-seq dataset color-coded by subtypes. **c**–**e** The semisupervised trajectory of all epithelial cells inferred by Monocle v2, color-coded by state (**c**) or subtypes **d** or stemness. Stemness levels were calculated as the mean expression of stem-like signature (**e**, **f**). Volcano plot showing differentially expressed genes between tumor cells and other normal epithelial cells (non-malignant cells). (*P*-value < 0.05, Wilcoxon rank sum test, log_e_ (fold change) > 0.25. **g** Significant biological processes (GO terms) enriched in tumor/malignant cells by clusterProfiler (hypergeometric test). **h** t-SNE plot of 3150 tumor cells derived from the CRC scRNA-seq dataset, color-coded by cell subtypes (**h**). **i**, **j** The trajectory of tumor cells inferred by Monocle v2, color-coded by cell subtypes (**i**) and sample origins (**j**).

It has been suggested that human colon cancer cells recapitulate the multilineage differentiation processes of normal colon epithelia. To investigate each lineage contributing to the CRC heterogeneity at single cell resolution, we subclustered cell populations for each cell type to identify subpopulations. To annotate these subpopulations, we combined another published CRC cohort consisting of 6 CRC patients in tumor regions as well as matched normal mucosa [29], and transferred the annotations of subtypes to our datasets in this study with the Seurat R package (Fig. 2a). Since the transition from normal epithelium to intraepithelial neoplasia were found to be associated with CRC patient survival [24], we focused on epithelial cells and found 9 subpopulations, namely CD19 + CD20 + B cells, crypt cells, enterocytes, goblet cells, intermediate, mature colonotypes, proinflammatory, stem-like, and tumor cells (Fig. 2b).

To distinguish malignant cells and nonmalignant cells in epithelial cells, we performed scRNA-seq-based copy number variation (CNV) and subclustering analysis (Fig. S2a, b). The proportions of malignant cells in each subcluster of epithelial cells were shown in Fig. S2c. The trajectory revealed a transcriptional hierarchy, defining seven molecular states (Fig. 2c, top). The cells from tumor tissues dominated the divergent differentiation states 2 and 5, suggesting the tissue arrangement along pseudotimes (Fig. 2c, bottom).

To illustrate the differentiation paths across the multilineages among the epithelial cell populations, the semisupervised trajectory inferred by monocle2 [33] revealed a transcriptional hierarchy defining three branches. The hierarchy was dominated by malignant epithelial cells, as well as normal epithelial cells (including goblet cells and (stem-like/transit amplifying cells) and immune-related cell types (including proinflammatory and mature colonotypes), which originated from normal epithelial cells with branching toward malignant epithelial cells (gray) and immune-related cell types (light green), respectively (Fig. 2d). Projection of malignant epithelial cells along the epithelial cell differentiation trajectory revealed segregation of tumor cells from normal epithelial cell types and stem-like populations. The greater stemness of malignant epithelial cells suggested the regenerative/proliferative potential of these tumor cells (Fig. 2e). The hypoxia and epithelial mesenchymal transition (EMT) were also investigated in the malignant epithelial cell populations (Fig. S2e).

### Transcriptional and functional features of malignant cells reveal heterogeneity in CRC patients

To characterize the malignant cell populations, we scrutinized the transcriptional features between malignant and nonmalignant cells. The known malignant epithelial cell populations characterized by upregulated expression of S100A4, VEGFA, MYC, and ICAM1 (intercellular adhesion molecule-1), according to their significant differential expression (log_e_|fold change | > 0.25, T test, *p* value < 0.05) (Fig. 2f, left). The most differentially expressed gene EMP3 (Epithelial membrane protein 3), which has been identified as an tumor suppressor in breast cancer [34], glioma [35] and remains to be elucidated in colon cancer. The characteristic genes in malignant epithelial cell populations were found to be involved in biological processes such as matrix remodeling, cell proliferation and apoptosis (Fig. 2f, right, Supplementary Table S6), hinting the occurrence of EMT. Moreover, the terms ‘positive regulation of angiogenesis’, ‘cellular response to decreased oxygen levels’ and ‘extracellular matrix organization’ were enriched with differentially expressed genes, also suggestive of the malignant tendency of the cell populations. Using genes that characterizing malignant epithelial cell populations, the biological pathways that those genes implicated in were shown in Fig. S2d. In addition, we predicted the regulons for malignant and non-malignant cells, respectively. The list of top 5 regulons for the two cell populations were shown in the heatmap (Fig. 2g). In malignant cells, a famous oncogene *MYC* was shown to be one of the key regulons. The transcriptional regulation role of *ATF1* in CRC cell lines has been characterized by a study of combing RNA-seq and ChIP-seq assays, in which found rs7017386 allele-specifcally enhanced the binding affnity of *ATF1* and promotion of two oncogenic lncRNAs via forming a long-range chromatin loop [36]. Therefore, both the characteristics of the Stemness, hypoxia, and EMT and the prediction of oncogenic regulons consistently reflected the malignancy and tumorgenesis roles of the identified malignant epithelial cells aforementioned.

To focus on transcriptional programs for subcategorizing tumor cells, we performed subclustering and trajectory analysis for malignant cell populations. We assigned seven tumor cell subclusters to all the malignant cell populations, namely tumor_CAV1, tumor_ATF3_JUN | FOS, tumor_ZEB2, tumor_VIM, tumor_WSB1, tumor_LXN, and tumor_PGM1 (Fig. 2h, Fig. S2f). The trajectory revealed a transcriptional hierarchy, converging into three discrete tumor subclusters. One subcluster was highly enriched with gene response to histone deacetylase (HDAC) inhibitors (like *ATF3* and *CAV1*), and another subcluster was highly enriched with inflammatory gene (*LXN* and *PGM1*). The subcluster enriched with tumor metastasis-related hypoxia (*WSB1*) originated from the aforementioned differentiation paths. Sample arrangement along the differentiation trajectory of tumor cells revealed malignancy in patient T0602.

### Transcriptional features in a spatial resolution on cryosections from patients with CRC

**Fig. 3:**
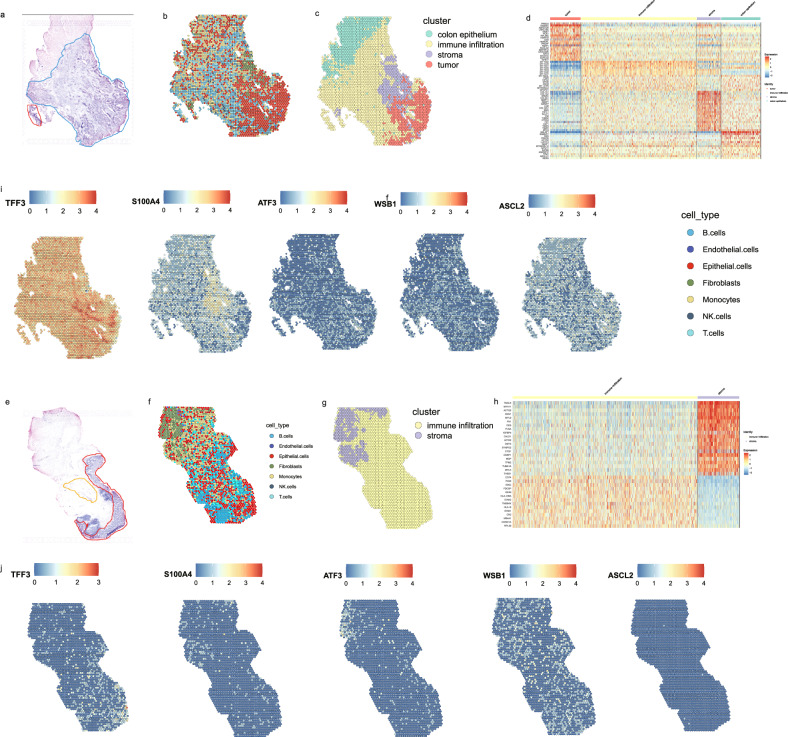
Spatial transcriptome (ST) of CRC and mapping of cell types at spatial resolution. **a** A pathologic section from tumor tissues of one CRC patient (T0602). **b** Annotations obtained by integration analysis of the CRC. scRNA-seq dataset and CRC5_1 in ST-seq dataset using seruat labeltransfer. **c** Clustering of the CRC5_1 ST spots and annotating CRC5_1 tumor cryosection on the ST slide. CRC5_1 cryosection was obtained from tumor tissues of patient T0602. **d** Expression levels for genes with subtype-specific patterns in CRC5_1 ST spots. **i** Standardized expression levels of five genes in the CRC5_1 in ST-seq datasets. **e** A pathologic section from normal, adjacent tissues of the CRC patient (T0602). **f** Annotations obtained by integration analysis of the CRC scRNA-seq dataset and CRC5N_1 in ST-seq dataset using seruat labeltransfer. **g** Clustering of the CRC5N_1 ST spots and annotating CRCN5_1 normal cryosection on the ST slide. CRC5N_1 cryosection was obtained from adjacent tissues of patient T0602. **h** Expression levels for genes with subtype-specific patterns in CRC5N_1 ST spots. **j** Standardized expression levels of five genes in the CRC5N_1 in ST-seq datasets.

To examine spatially transcriptional differences within colorectal tissues, we mounted cryosections of unfixed CRC tissues originating from the same CRC patients onto spatially barcoded ST microarray slides to generate unbiased transcriptome maps. After haematoxylin and eosin (H&E) staining and brightfield imaging, we annotated the slides according to the distinct histological features (Fig. 3a). The samples were then processed for ST analysis. We demultiplexed the sequenced reads and identified their spatial location within tissues using the ST location-specific barcodes of the array. For patient T0602, we detected approximately/average 3413 median UMIs and approximately/average 1660 median gene numbers per ST spot for both the tumor tissue section (named CRC5_1) and the normal tissue section (named CRCN5_1).

First, the spatial transcriptomics data were integrated with the scRNA-seq data using Seurat-v3 anchor-based integration to annotate each region in the corresponding section [37, 38]. Every spot in the spatial data was considered a weighted mix of cell-types identified by scRNA-seq. For each spot, the cell type with the maximum prediction score among all possible cell types and thus transferred from the scRNA-seq dataset is illustrated (Fig. 3b, f). After further adjustment on the basis of annotated histological features, we annotated four and two anatomical regions in the CRC5_1 section (derived from a tumor tissue, Fig. 3c), and in the CRCN5_1 section (derived from an adjacent tissue), separately (Fig. 3g). We observed many obviously characteristic genes, which represented higher expression in annotated regions especially in the tumor tissues (Fig. 3i) compared to normal tissues (Fig. 3j). It is noted that five DEGs in comparision of malignant and non-malignant cells in the CRC scRNA-seq dataset were included IFITM1, CXCL1, CXCL8, S100A4, and TGFBI. The higher expression in tumor or stromal regions were shown in Fig. S3. *IFITM1* was highly expressed and spatially restricted relative to the annotated tumor regions. *IFITM1* is an interferon-induced transmembrane protein family member. The roles of *IFITM1* has been summarized that it involves in gallbladder carcinoma, esophageal adenocarcinoma, colorectal cancer, and gastric cancer [39]. Fang et al. investigated that over-expression of *IFITM1* promoted the aggressiveness of CRC cells, whereas knockdown of *IFITM1* expression inhibited cell migration, invasion or tumorigenicity in vitro [40]. Pauline et al. found a highly significant increase in *IFITM* mRNA levels in 154 patients with colon and rectal carcinomas, compared to corresponding normal tissues [41]. Kelemen et al. thought that *IFITM1* expression determined extracellular vesicle uptake in colorectal cancer [42]. *CXCL1* and *CXCL8*, members of the angiogenic CXC chemokine family are highly expressed only on the border of annotated tumor regions in our study, suggesting that an immune exclusion and blockage by the tumor. *S100A4* (S100 calcium-binding protein A4) was a typical feature in the annotated stromal region in our study. Angiogenesis and prognostic roles of *S100A4* in colorectal cancer have been investigated [43, 44]. *TGFB1* is highly expressed and spatially restricted relative to the annotated stromal region in our study. *TGFB* is released by macrophages and fibroblasts, and it modulates cell growth, differentiation, and cell death in colorectal cancer [45]. TGFB signaling is implicated in metastasis of colorectal cancer [46]. In addition, the genes characterizing classical phenotypes of cancer stem-like (*ASCL2*), hypoxia (*WSB1*) and apoptosis (*ATF3*) were all highly expressed in the tumor or stromal region, suggesting cell differentiation programs. To facilitate comparisons, the expression of the aforementioned genes in the adjacent normal tissue samples is presented (Fig. 3j).

### Different anatomical regions in a spatial resolution represents cell compositions on tumor, stroma, immune and epithelium

Second, standalone analysis of the spatial transcriptome along with the annotated anatomical regions was performed to identify spatially differential transcriptional programs. Interestingly, we found region-specific transcriptional differences. Unsupervised clustering of spatial transcriptome of CRC5_1 along with annotated anatomical regions revealed that the tumor region was characterized by heterogeneous cell cluster enriched high TMSB4X expressing cell populations (Fig. 3d, Fig. S3), which was not observed in CRCN5_1 (Fig. 3f). The higher expression of *TMSB4X* is consistent with the recent finding that *TMSB4X* is one of the markers of epithelial cells in CRC tumor tissue [47]. *TMSB4X* encodes thymosin beta-4, a well-known secreted small peptide, which is identified as a novel prognostic marker for non-small cell lung cancer [48]. *TMSB4X* has been validated as a therapeutic target in colon cancer stem cells in a previous study [49]. Additionally, in other architecture-dependent tissues whose spatial locations are deeply intertwined with their functions, such as brain and heart, *TMSB4X* has been reported to be involved in tumor progression via neovascularization, cell adhesion and the epithelial-mesenchymal transition [50, 51]. When TMSB4X was silenced both in vitro and in vivo, differentiation and tumorigenicity were diminished [52]. Targeting highly expressed TMSB4X or TMSB4X-high cell populations identified in spatial trascriptome in CRC has the potential to become a new therapeutic strategy in CRC. Moreover, the annotated stromal region overexpressed classical marker genes for fibroblasts or endothelial cells, such as *LUM*, *VIM*, *COL1A1*, and *COL1A2*. It is noted that the differential expression of *VIM* gene in comparison of stromal regions and other regions in CRC ST-seq dataset was also identified in CRC scRNA-seq dataset, in which *VIM* gene was one of the DEGs in malignant cells compared to non-malignant cells.

**Fig. 4:**
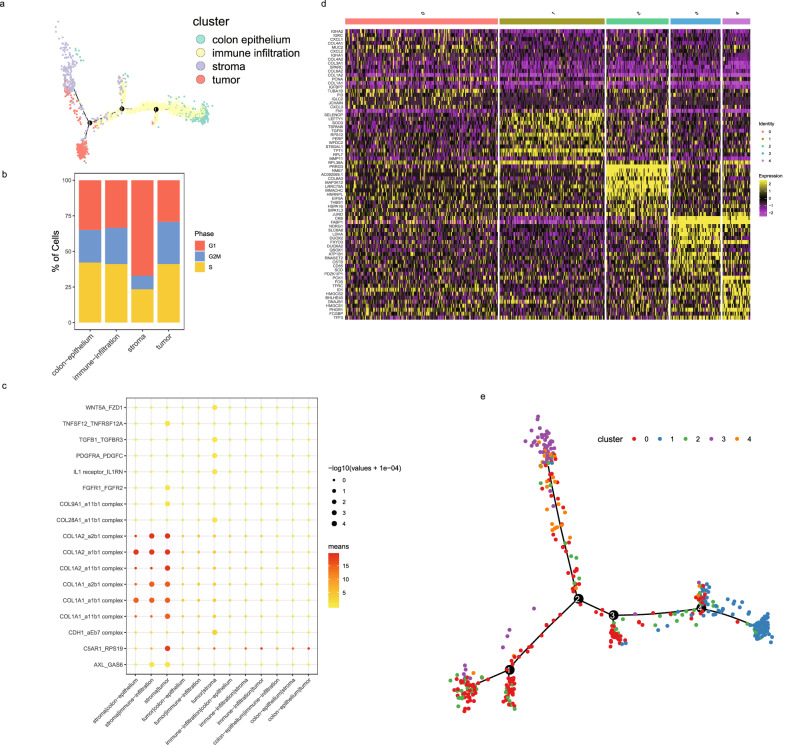
The trajectory and interactions of cell lineages at spatial resolution. **a** The trajectory of all ST spots of CRC5_1 cryosection in the ST-seq dataset, color-coded by four annotated, spatial regions (tumor, stroma, immune infiltration, colon epithelium, as shown in Fig. 3c. **b** The distribution of ST spots of CRC5_1 cryosection on cell cycle phases. **c** Receptor-ligand pair expression in each pair of spatial regions in CRC5_1 cryosection using CellPhoneDB. **d** Hierarchical clustering of ST spots from the tumor region in CRC5_1 cryosection and indicative of five tumor subtypes based on five transcriptomic signatures. **e** The trajectory of five tumor subregions.

As proximity is a necessity for physical interactions among cells, anatomical regions or cell-type proximity/interactive maps can be used to guide the discovery of interactions between anatomical regions or cell types in the same or different lineages. We first carried out a pseudotemporal trajectory analysis of the four anatomical regions in CRC5_1 section. Our data revealed that the lineage stemmed from the colon epithelium, and then went through an infiltration to divide into two major lineages, corresponding to stromal and tumor spots, respectively (Fig. 4a, top). The trajectory reconstruction confirmed two major terminal/branching cell fates at tumor and stoma spots, respectively (Fig. S4a). Moreover, seven continuous states were identified during along pseudotime trajectory (Fig. S4b). Tumor spots were observed mainly in state 5, and stromal spots were found primarily in state 6, which indicated different states during tumorgenesis, even though these two anatomical regions were proximal to each other. The colon epithelium was observed mostly in state 1, and immune infiltration was distributed in states 2,3,4 and 7, sporadically in state 6.

Consistent with the trajectory, the tumor and stromal spots exhibited greatest pseudotime meaning the most extent of differentiation and mature/terminal programme (Fig. S4b). To explore the characteristics of the branching cell fates, cell cycle phase analysis was performed. Approximately 70% of stromal spots arrested in the G1 phase of cell cycle, but this outcome was not observed in tumor anatomical regions (Fig. 4b). G1 phase cell cycle arrest may be responsible for the inhibition of colorectal cell proliferation in the stromal region. Compounds that promote G1 cell cycle arrest were used and confirmed to be a treatment of colorectal cancer [51].

### Spatially resolved interactions of tumor and stromal regions

To investigate the regulators critical for the branching cell fates of tumor and stromal regions, SCENIC-based regulon analysis was performed. The results showed a spatial-resolved, specific regulon set (Fig. S4c). Four of the top 5 regulons in stromal or tumor region were overlapped, including *CDX1*, *IRF8*, *HNF4A*, and *CREB3lL1* (Fig. S4d). The stromal region was specifically regulated by *IRF3*. The previous research suggested that overexpression of *IRF3* causes cell-cycle arrest in the G1/S phase thereby resulting in inhibition of DNA synthesis [53]. However, the tumor region was found to be specifically regulated by *POU2F2*. A recent research suggested that *POU2F2* played tumorigenic roles in glioblastoma by leading to a metabolic shift towards aerobic glycolysis [54], but the roles in colorectal cancer remain poorly understood. In conclusion, the transcription factor *IRF3* plays a key role in the generation of stromal cells in the stromal region, but not tumor cells in the tumor region, and the underlying programme involves in inhibition of cell proliferations by arresting cells in the G1/S phase.

To further investigate the interaction between the neighboring anatomical region, especially between the tumor region and other regions, we secondly performed CellphoneDB-based cell interaction analysis to investigate the underlying ligand-receptor pairs in different anatomical regions derived from CRC tumors. The aforementioned trajectory suggested the crosstalk between stromal region and tumor anatomical region (Fig. 4a, top). We observed intensive cellular interactions between the stromal region and tumor regions. (Fig. 4c). For example, the stromal region was predicted to interact with the tumor region in tumor tissues through C5AR1-RPS19, which is known to promote tumor growth by facilitating recruitment of these cells to tumors [55]. *C5AR1* is known to activate and recruit myeloid-derived suppressor cells to tumors and reshape immunosuppressive tumor microenvironment [56]. Accordingly, research studies have shown that the activated complement system has a tumor-promoting effect, including angiogenesis, trophoblastic invasion and tissue remodeling, which includes processes favorable for tumor establishment and progression [57, 58]. It was proposed that *RPS19* was one of the marker of epithelial-mesenchymal transition (EMT), and regulated the metastasis abilities of cancer cells by in vitro assays [59]. Decreasing *RPS19* in tumor cells or interrupting the C5AR1-RPS19 interaction reduces RPS19-mediated immunosuppression, impairs tumor growth, and delays the development of tumors in an in vivo assay of breast cancer [60].

To evaluate the refined characterization of the tumor region, we reclustered the spots in the tumor region to discern any spatial differentiation process. Reclustering the spots in the tumor region led to the identification of five subregions corresponding to five gene modules (Fig. 4d): C0 spots expressed high levels of collagen, which is the major component of the TME and participates in cancer fibrosis [61], including collagen type III (*COL3A1*), *COL4A2*, *COL4A1*, *COL6A2*, *COL1A2*, and *COL1A1*. In contrast to C1 spots, C0 spots in the outermost layer in the CRC5_1 section, constituted the primary structural element of the ECM. C1 spots expressed high levels of ECM transcripts implicated in cell migration, including fibronectin (FN1), tumor protein, transnationally-controlled 1 (TPT1), transforming growth factor beta induced (TGFBI), and whey acidic protein (WAP) 4-disulfide core domain protein 2 (WFDC2). In addition, C1 spots maintain an intermediate phase with both undifferentiated and differentiated phenotypes. Transferring cell type labels to spatial data suggested that only the epithelial cells in scRNA-seq were spatially restricted to the outermost layer of the section (tumor subcluster C0), and C1 was spatially restricted to the center of the section. C2 spots expressed extremely high levels of proline rich and Gla domain 3 (PRRG3), which is a member of a family of vitamin K-dependent transmembrane proteins that contain a glutamate-rich extracellular domain. C2 spots characterized by a senescence-like proliferation, are represented by MAP3K12 [62]. C3 spots expressed extremely high levels of fatty acid-binding protein 1 (FABP1), which is essential for proper lipid metabolism in differentiated enterocytes [63]. The creatine kinase B (CKB) promotes metastatic survival by modulating intra- and extracellular energetics [64]. C3 spots are characterized by metabolism. C4 spots expressed high levels of mitochondrial phosphoenolpyruvate carboxykinase 1 (PCK1) gene, which increases colon cancer cell growth in part by promoting the consumption of both glucose and glutamine in the tricarboxylic acid (TCA) cycle [21]. The roles of hypoxia-reprogrammed TCA cycles in promoting human breast cancer cell growth via a HIF-1α-mediated PCK2 pathway have been reported [65]. C4 spots are characterized by hypoxia-response.

Based on characteristic genes of five subregions of tumor region, we defined five gene modules, including focal adhesion dynamics (C0), intermediate (C1), ECM (C2), metabolic (C3), hypoxia-response (C4) modules. The results suggested roles for these gene modules in tumor progression, implying a need for a combination of an anticancer therapy with corresponding modulators.

## Discussion

Colorectal cancer is a complex and heterogeneous malignant tumor of the colon and rectum. According to the degree of tumor differentiation and invasion, CRC can be classified into different subtypes. However, the published CRC studies usually were performed with whole tissues, such as bulk RNA-seq, blurring the heterogeneous characteristics of different cell types and limiting the ability to capture tumor heterogeneity. Cancerous tissue is composed of a mixture of various components, such as tumor cells, stromal cells, immune cells, and ECM, leading to a complex TME [66]. The TME components exhibit interactive crosstalk with tumor cells and their surrounding factors, which in turn shapes tumor structure, metabolism, and secretion, thus affecting tumor development and/or metastasis. Immune cells within the TME play crucial roles during tumorgenesis. Immunotherapy aims to fight against cancer, infection, and other diseases stimulating or suppressing the immune system. Immunotherapy displays promising therapeutic outcomes and limited side effects [67]. An increasing number of clinical trials have proven the effects of immunotherapy in certain types of solid tumors, such as melanoma, non-small-cell lung cancer, renal cancer, and prostate cancer [68]. Recently, immunotherapy drugs such as CAR-T drugs have been approved by the FDA for clinical application. However, not all patients respond favorably to immunotherapy. Researchers have begun to examine the complexity and diversity of the TME and are realizing its importance in immunotherapy. Focusing on the TME facilitates to better understanding of the occurrence, development and metastasis of tumor and may lead to better diagnosis and treatment [69]. The expression pattern and function of tumor cell-associated immune molecules from in the TME provide useful information to determine whether a patient will might benefit from immunotherapy. There is an urgent need for improved techniques to better understand the TME and analyze the composition of immune cells and various other various cell types in tumor tissues. Single-cell sequencing and spatial transcriptomics have satisfied this requirement, and these two new-newly emerging techniques can be used to analyze samples at the single-cell level and monitor the in situ spatial information of tumor tissues.

Single-cell RNA sequencing enables investigation of the transcriptional regulation of highly heterogeneous cell populations or subpopulations and facilitates the discovery of genes that indicate cell subtypes, or that mark intermediate states during a biological process, as well as bifurcation between two alternative cellular fates. Spatial RNA sequencing enables the anatomical, in situ locations to be preserved, but cannot achieve rigorous single cell resolution. Actually, 10–20 cells are typically identified in each spot in the ST assay. Since in situ locations representing tissue sections are lacking in the scRNA-seq and single-cell resolution is lacking in the spatial RNA-seq, it is necessary to combine single-cell RNA-seq and spatial RNA-seq to reflect both cellular locations at true single-cell resolution. This allows inferences on functional relationships between scRNA-seq-defined populations based on their colocalization in space, and ultimately provides a more comprehensive characterization of cell types in their native environment than can be gained from either modality alone. In addition to transferring annotation at single cell level to spatial locations, we think that the current combinatory analysis between single-cell and spatial transcriptome remains to be more closely and complex.

In this study, we combined single-cell and spatial transcriptomics to create a hierarchical map of cellular lineages in CRC. There CRC patients are involved, as well as single-cell transcriptomes of ~47,000 cells, and spatial gene expression maps. We first constructed CRC single-cell maps consisting of epithelial cells, mast cells, monocytes, T cells, B cells, Endothelial cells, and NK cells. Then we focused on the epithelial cells, from which we identified malignant cells. Also, we performed analyses of differential expression, functional enrichments, transcription factors to characterize the features of malignant cells compared to non-malignant cells. By sub-classification of malignant cells, we found seven subtypes according to transcriptional features, which could be helpful to molecular subtyping of CRC. Combining spatial and scRNA-seq datasets using bioinformatics approaches, we transferred cell type annotations at single cell level to those at the spot level. The results showed four regions including tumor, stroma, immune-infiltration, and colon epithelium. Then we mainly focused on proximate cellular interactions (tumor and stroma) within colorectal tissue, to quantify anatomically restricted gene expression and explore crosstalks between tumor and stromal regions. Tumor and stromal regions were characterized by TMSB4X and VIM high expressions, respectively. The cellular interactions or crosstalks were inferred to be mediated by C5AR1 and RPS19, which remained to be further validations.

In summary, we comprehensively explored the cellular landscape and reconstructed the putative interaction network consisting of tumor cells and their microenvironment. This collective view allowed us to elucidate how diverse cellular components jointly determine CRC molecular subtypes in individual patients.

## Materials and methods

### Subjects and clinical characteristics

We chose the patient inclusion criteria as the clinical stage of the tumor being stage 2 and stage 3, without the presence of intestinal obstruction or abdominal infection. Three patients were included and all patients were treatment-naive before tumor resection. No one knows the underlying mechanism heterogeneity in a single cell level. Matched adjacent normal tissues and primary tumors and peripheral blood were obtained from all 3 patients (CRC0529, CRC0602, CRC0609). The detailed clinical information were shown in the Supplementary Table S7. All sampling and experimental steps in this study were approved by the Ethics Committee of Zhuhai People’s Hospital Affiliated with Jinan University (Research projects IRB Review Approval Notice: LW-[2022]#1). Relevant informed consent documents were signed by the participants before sample collection and data acquisition, all participants received no compensation from this study.

### Preparation of single-cell suspensions

All tissue samples were washed twice with cold PBS. Tissue samples were cut into 1 mm^3^ in size and placed in petri dish with cold PBS, then transferred into centrifuge tube, adding appropriate amount of enzyme and shaking at a certain temperature for a period of time. After 2-3 minutes’ standing, supernatant were collected and then use a filter membrane to remove large clumps. After centrifuge the cells were collected, and then we resuspended the cells with red blood cell lysis buffer and incubate 2-3 min at room temperature and then centrifuge at 120×g under 4°C for 3 min. Samples were resuspended again with cold PBS.

### Droplet-based single-cell sequencing

Using the Single Cell 5’ Library and Gel Bead Kit (10X Genomics, 120237) and Chromium Single Cell A Chip Kit (10X Genomics, 120236), the cell suspension was loaded onto the Chromium single-cell controller (10X Genomics) to generate single-cell gel beads in the emulsion (GEMs) according to the manufacturer’s protocol. Briefly, single cells were suspended in PBS containing 0.04% bovine serum albumin. Approximately 10,000 cells were added to each channel, and about 6000 cells were recovered. The captured cells were lysed, and the released RNA was barcoded via reverse transcription in individual GEMs. Reverse transcription was performed at 53°C for 45 min, followed by 85°C for 5 min, and then the temperature was held at 4°C in a C1000 Touch Thermal Cycler (Bio Rad). After reverse transcription, single-cell droplets were broken and the single-strand cDNA was isolated and cleaned with Cleanup Mix containing DynaBeads (Thermo Fisher Scientific). cDNA was generated and amplified, and quality was assessed using the Agilent 4200. Single-cell RNA-seq libraries were prepared using Single Cell 5’ Library Gel Bead Kit V2 following the manufacture’s introduction. Next generation sequencing was performed on an Illumina Novaseq6000 with a sequencing depth of at least 100,000 reads per cell and pair end 150 bp (performed by CapitalBio Technology, Beijing).

### Single cell RNA-seq (scRNA-seq) data processing

Sequencing data were aligned to the human reference genome (GRCh38) and processed using the CellRanger (version 4.0.0). The gene expression matrix from the CellRanger pipeline was filtered, normalized using the Seurat R package (v3.2) [37]. Cells were selected if they met the following criteria: (i) top 99% of cells in unique molecular identifier counts; (ii) >200 genes; and (iii) <25% of mitochondrial gene expression in UMI counts. After the removal of low-quality cells, the gene expression matrices were normalized to the total UMI counts per cell and transformed to the natural log scale. Then all the datasets of individual sample were integrated using the “FindIntegrationAnchors” and “IntegrateData” function in Seurat. Louvain algorithm was applied to iteratively group proximal cells together by “FindClusters” function with resolution of 0.6. Visualization was achieved by both the t-Distributed Stochastic Neighbor Embedding (tSNE) projection and Uniform Manifold Approximation and Projection (UMAP).

Cell type annotations were performed on Blueprint and Encode reference dataset via SingleR [70], along with the marker-based correction. We classified all cells into eight major cell types, including T cells, B cells, NK cells, Monocytes, Epithelial cells, Fibroblasts, Endothelial cells, and Mast cells.

### 10x Visium Spatial transcriptomics (ST)

Cryosections were cut at 10-μm thickness, mounted onto the GEX arrays. Sections were placed on Thermocycler Adaptor with the active surface facing up and incubated for 1 min at 37°C, and fixed for 30 min with methyl alcohol under −20°C, and then stained with H&E (Eosin, Dako CS701, Hematoxylin Dako S3309, bluing buffer CS702). The brightfield Images were taken on a Leica DMI8 whole-slide scanner at 10× resolution.

Visium spatial gene expression was processed using Visium spatial gene expression slide and Reagent Kit (10× Genomics, PN-1000184). For each well, Slide Cassette was used to create leakproof wells for adding reagents. 70 μL Permeabilization enzyme was added and incubated at 37 °C for 20 min. Each well was washed with 100 μL SSC, and 75 μL reverse transcription Master Mix was added for cDNA Synthesis.

cDNA library were prepared for sequencing. After the first-strand synthesis finished, reverse transcription Master Mix was removed from the wells, and then 75 μL 0.08 M KOH was added and incubated for 5 min at room temperature, then we removed the KOH from wells and washed with 100 μL EB buffer. A total of 75 μL Second Strand Mix was added into each well for second-strand synthesis. cDNA amplification was performed on a S1000TM Touch Thermal Cycler (Bio Rad). According to the manufacture’s introduction, Visium spatial libraries were constructed using Visium spatial Library construction kit (10× Genomics, PN-1000184). The libraries were sequenced using an Illumina Novaseq6000 sequencer with a sequencing depth of at least 100,000 reads per spot with pair-end 150 bp (PE150) reading strategy (performed by CapitalBio Technology, Beijing).

### Spatial transcriptome sequencing (ST-seq) data processing

The sequencing reads were mapped to the GRCh38 human genome and expression was quantified with the spaceranger-1.0.0. Further analysis was performed with Seurat (version 3.0.2). To annotate spots, we applied the integration workflow introduced in Seurat v3, which enabled the probabilistic transfer of cell types from the scRNA-seq data to the ST data. Specifically, we first identified pairwise correspondences between single cells and single spots to quantify the batch effect. Each spot was then annotated based on the transcriptomic similarity between spots and cell types in the scRNA-seq dataset. This probabilistic transfer procedure was implemented using the FindTransferAnchors (dims=1:30) and TransferData (dims=1:30) functions in Seurat with the combination of top 100 DEGs of each cell type.

### Differential expression and functional enrichment analysis

After dimensional reduction and projection of all cells into two-dimensional space by tSNE and UMAP, cells were clustered together according to common features. The “FindAllMarkers” function in Seurat was used to find markers for each of the identified clusters. Using differentially expressed genes (DEGs) of each cluster, we performed functional enrichment analysis which were implemented by clusterprofiler (v3.10.1) with |log2Foldchange | >0 and p.adj < 0.05 as thresholds (hypergeometric test). The enrichment analysis of comprehensive functions including Gene Ontology (GO), Kyoto Encyclopedia of Genes and Genomes (KEGG) pathways, Reactome and Disease. Gene sets enrichment analysis was performed by GSEA application version of JAVA (v2.2.2.4), which used predefined gene sets from the Molecular Signatures Database (MSigDB, v6.2).

### Regulon analyses

Regulon scores for individual cells were computed using the SCENIC (single-cell regulatory network inference and clustering) pipeline [71]. A log-normalized expression matrix of neuronal cells was used as an input into the pySCENIC workflow (https://pyscenic.readthedocs.io/en/latest/index.html) with default settings to infer regulons (master TFs and their target genes).

### CNV estimation and identification of malignant cells

The chromosomal CNA profile of single cells was inferred by the R package inferCNV (version 1.0.4) [72]. Average signal were used as reference to define a baseline of normal karyotype such that their average copy number value was subtracted from all cells. The following parameters were applied: cutoff=0.1, cluster_by_groups=TRUE, HMM = TRUE, and denoise=TRUE.

### Cell-cell communication analysis

In order to explore cell-cell communications via ligand–receptor interactions, we employed the strategy proposed by Vento-Tormo et. al. [73] based on a public repository of ligands, receptors and interactions database CellPhoneDB (v2.0) [74]. The interaction score between two different cell types was mediated by a specific ligand-receptor pair based on the mean gene expression of ligand from one cell type and the corresponding receptor from another cell type. To identify the significant cell-cell interaction, we permuted the change of cell type label for each cell at 1,000 times to calculate the significance of each pair (p-value < 0.01). This procedure was performed between all pairs of cell types. The interactions between distinct cell subpopulations via putative ligand-receptor pairs were visualized using the ggplot2 package.

### Single-cell trajectory analysis

We used Monocle v.2 [33] to illustrate the cell state transition in total epithelial cells, tumor cells in the CRC scRNA-seq dataset and in CRC5_1 tumor cryosection in the ST-seq dataset. This R package applied a reversed graph embedding technique to reconstruct single-cell trajectories. UMI count matrices and the negbinomial.size parameter were used to create a CellDataSet object in the default setting. We filtered variable genes with the following cutoff criteria: (1) genes expressed in more than 10 cells; (2) average expression value > 0.1; and (3) Qval < 0.01. These variable genes were used for semisupervised trajectory reconstruction. Dimensional reduction and cell ordering were performed using the DDRTree method and the orderCells function.

### Supplementary information

Supplementary material file legend

Figure S1.

Figure S2.

Figure S3.

Figure S4.

Supplemental tables

## Data Availability

The raw sequence data reported in this study have been deposited in the Genome Sequence Archive [75], at the National Genomics Data Center, Beijing Institute of Genomics, Chinese Academy of Sciences/China National Center for Bioinformation (BioProject Accession: PRJCA022899, GSA: HRA009354), and are publicly accessible at http://bigd.big.ac.cn/gsa. The additional data that support the findings of this study are available from the corresponding author on request.
