# Supplementary material for: Integrating spatial and single-cell transcriptomics reveals tumor heterogeneity and intercellular networks in colorectal cancer
Source: Cell Death Dis. 2024 May 10;15(5):326. doi: 10.1038/s41419-024-06598-6 (PMC11087651; doi:10.1038/s41419-024-06598-6)
Supplement: Supplementary file 1 — Supplementary material file legend [file 41419_2024_6598_MOESM1_ESM.docx]

**Supplementary files**

Figure S1. The annotations and cell proportions of eight cell type in CRC scRNA-seq.

Figure S2. The transcriptomic and functional features of malignant cells identified from epithelial cells.

Figure S3. The spatial distribution of typical genes on the cryosections from patients with CRC.

Figure S4. The trajectory and regulons of four spatial regions on the cryosections from patients with CRC.

Table S1. Gene and UMI of each patient and cryosections.

Table S2. Cell number information of each major cluster.

Table S3. All upregulated-gene of each major cluster.

Table S4. Major clusters distribution in CRC and normal tissues.

Table S5. Major clusters distribution in CRC and normal tissues of each patient.

Table S6. The list of enriched GO terms using DEGs of malignant cells compared to non-malignant cells.

Table S7. Patient samples and clinical characteristics.
