# Supplementary figures and images for: Integrating spatial and single-cell transcriptomics reveals tumor heterogeneity and intercellular networks in colorectal cancer
Source: Cell Death Dis. 2024 May 10;15(5):326. doi: 10.1038/s41419-024-06598-6 (PMC11087651; doi:10.1038/s41419-024-06598-6)

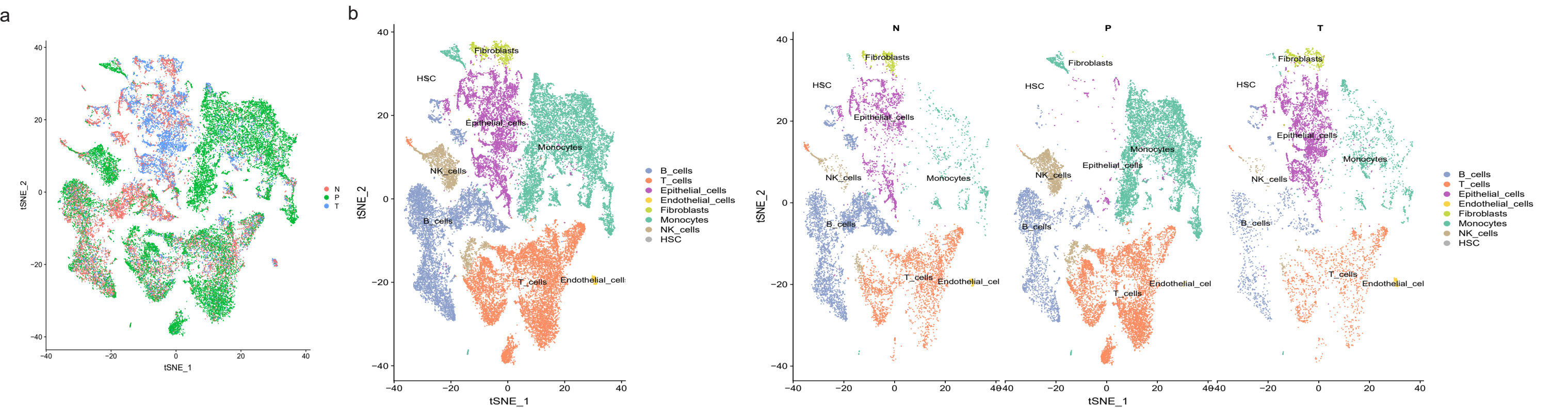

Supplement: Supplementary file 2 — Figure S1. [file 41419_2024_6598_MOESM2_ESM.pdf]

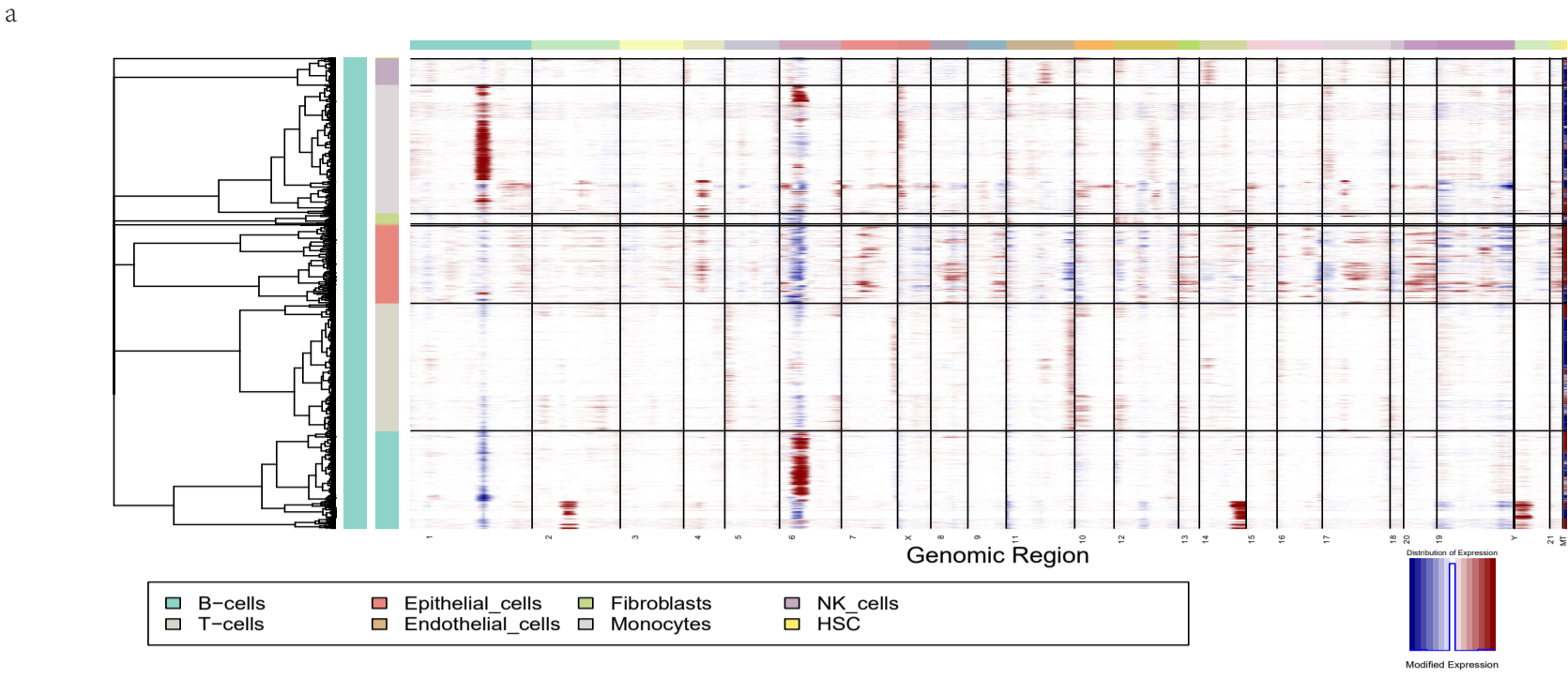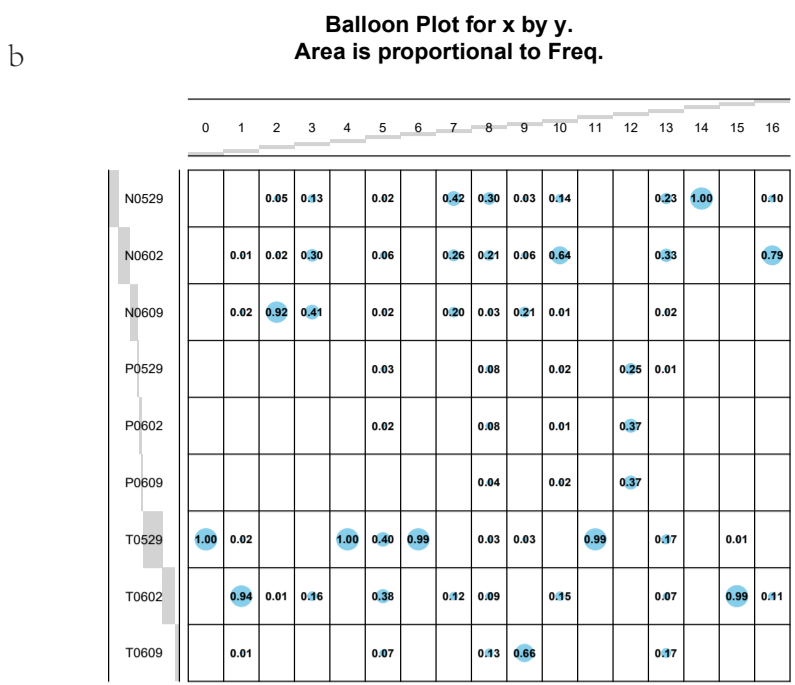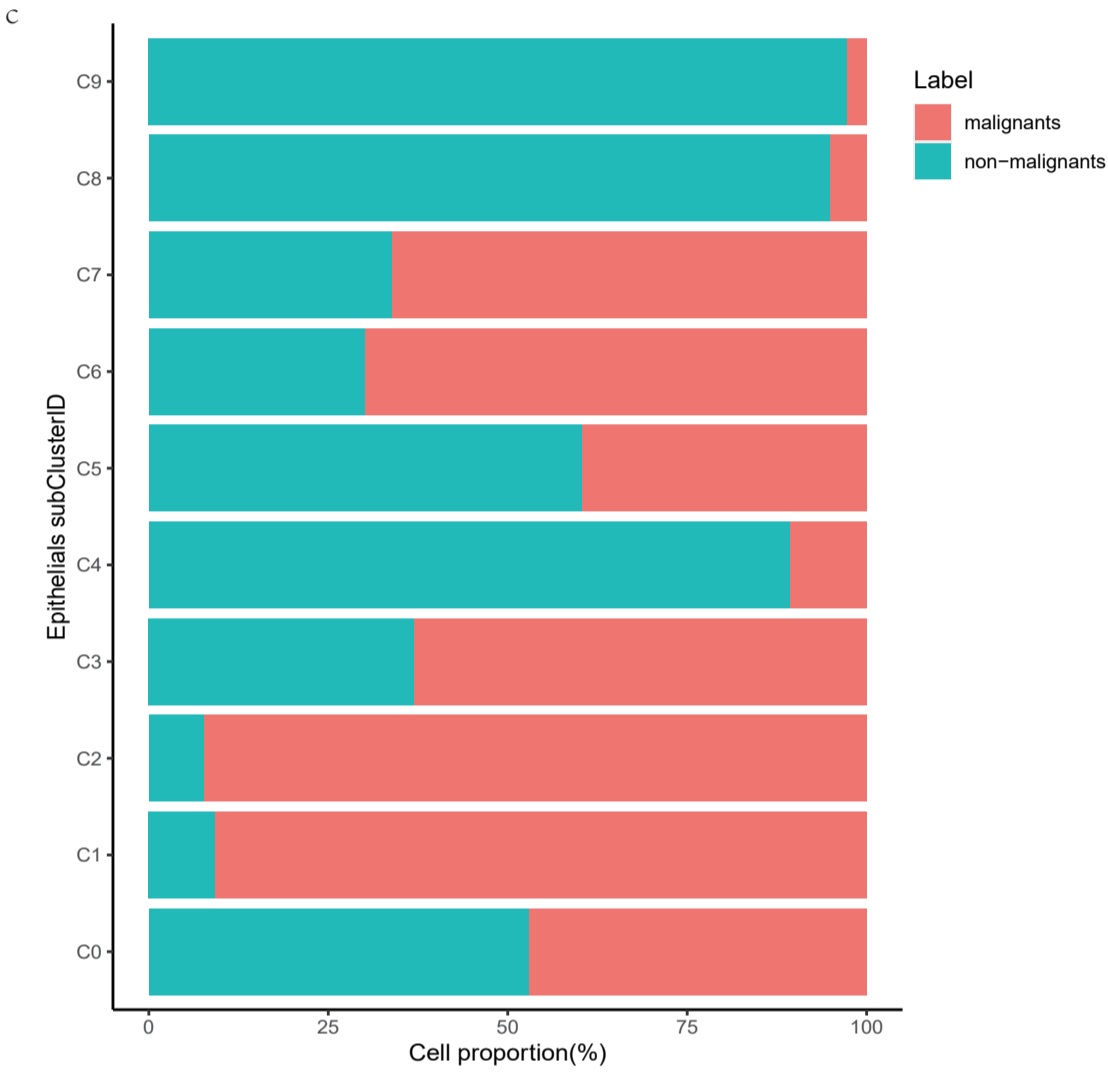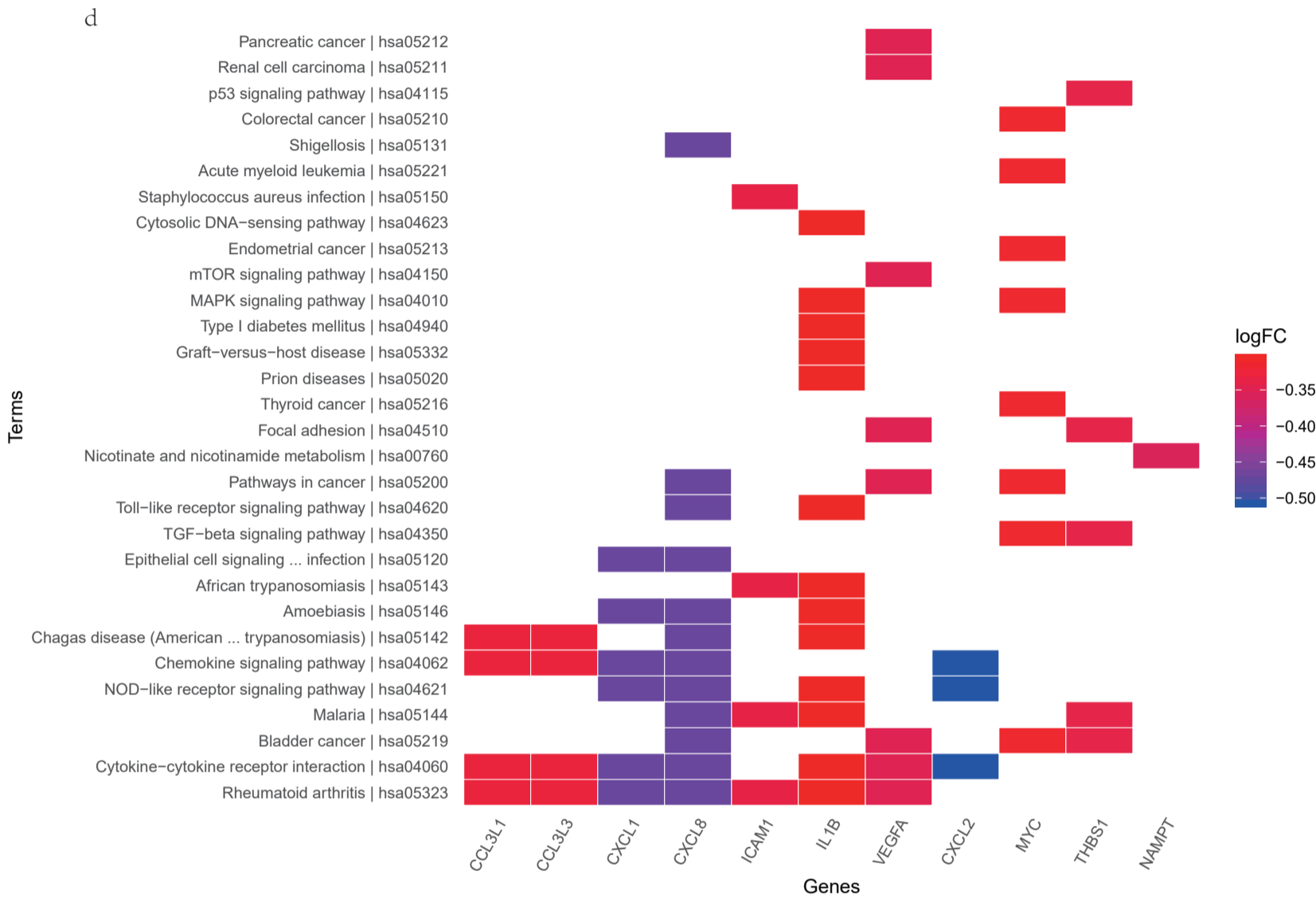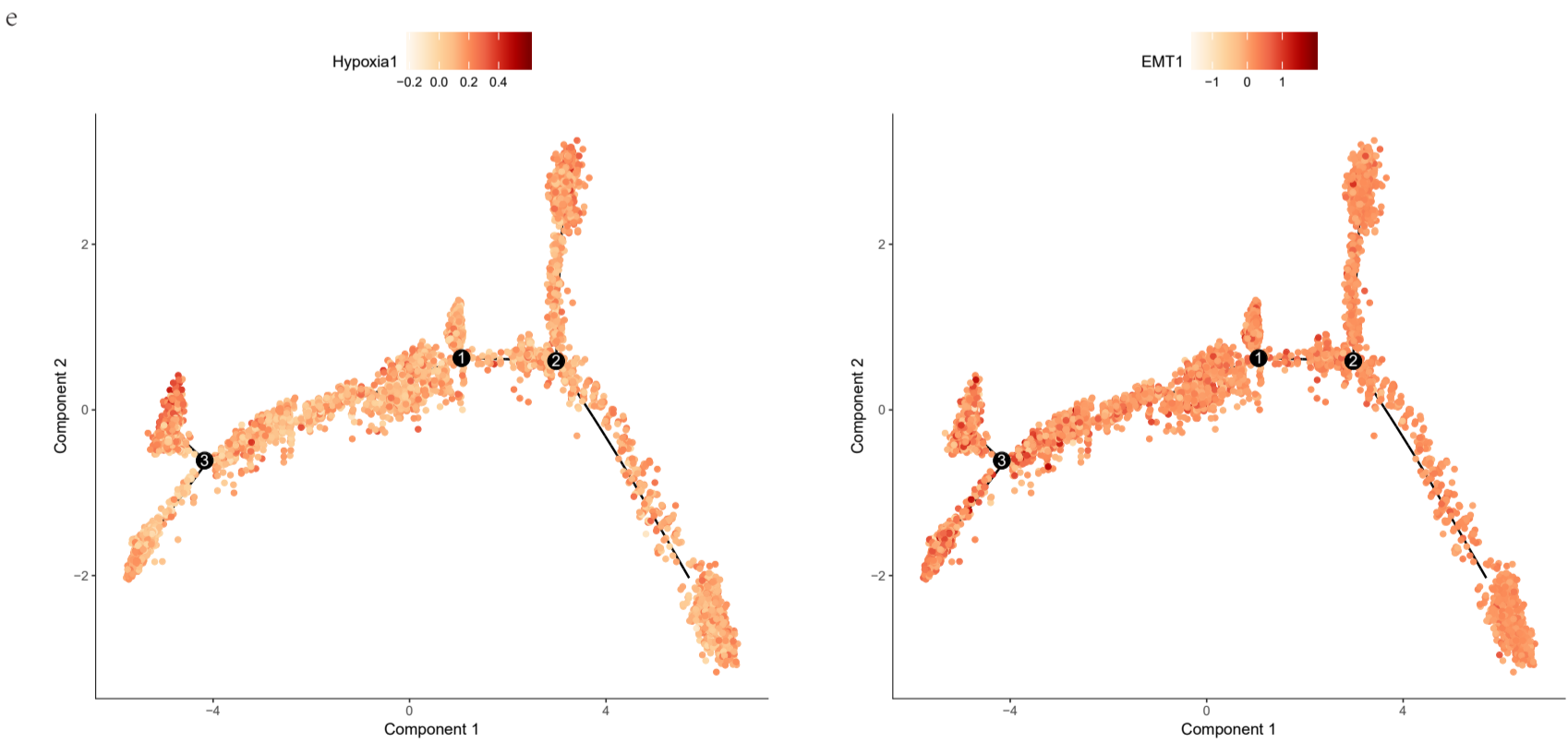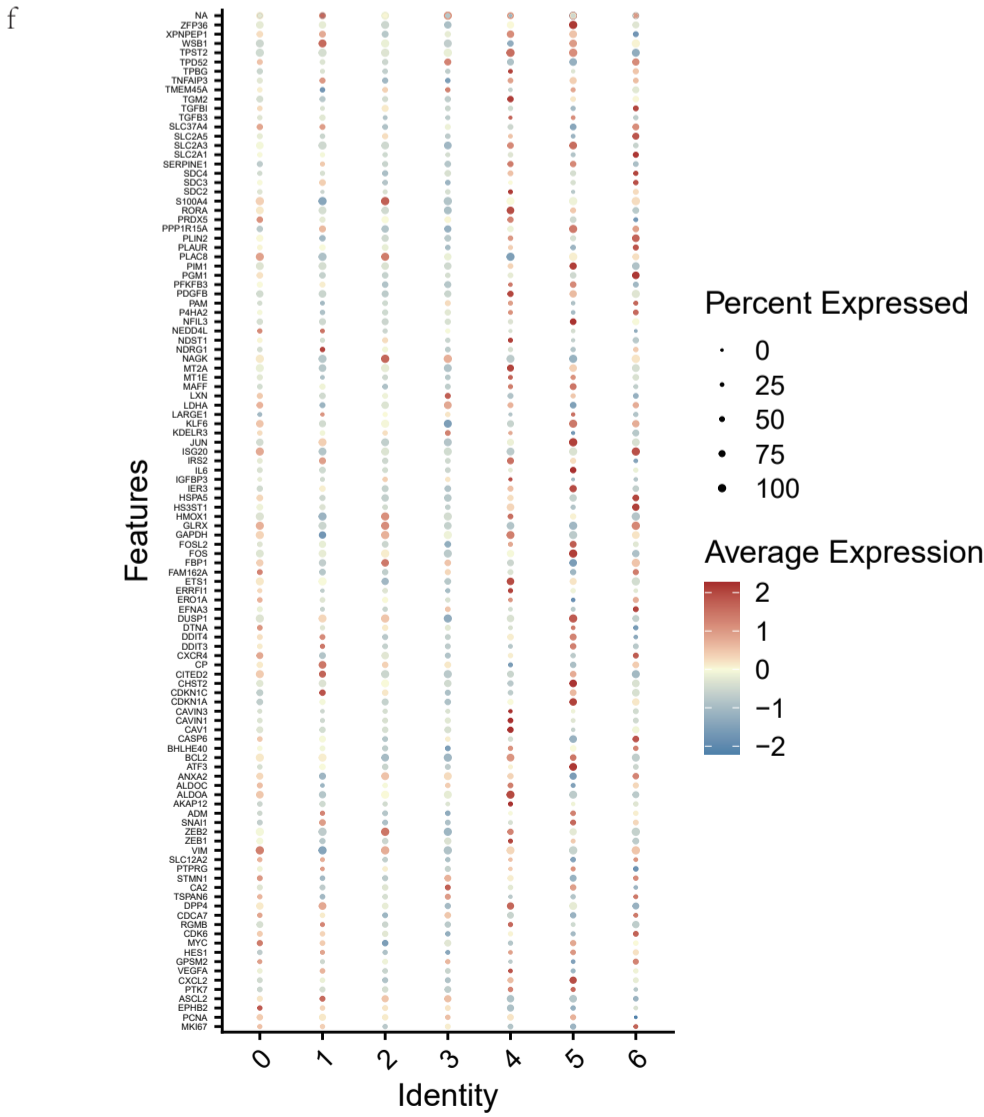

Supplement: Supplementary file 3 — Figure S2. [file 41419_2024_6598_MOESM3_ESM.pdf]

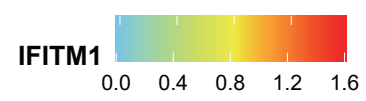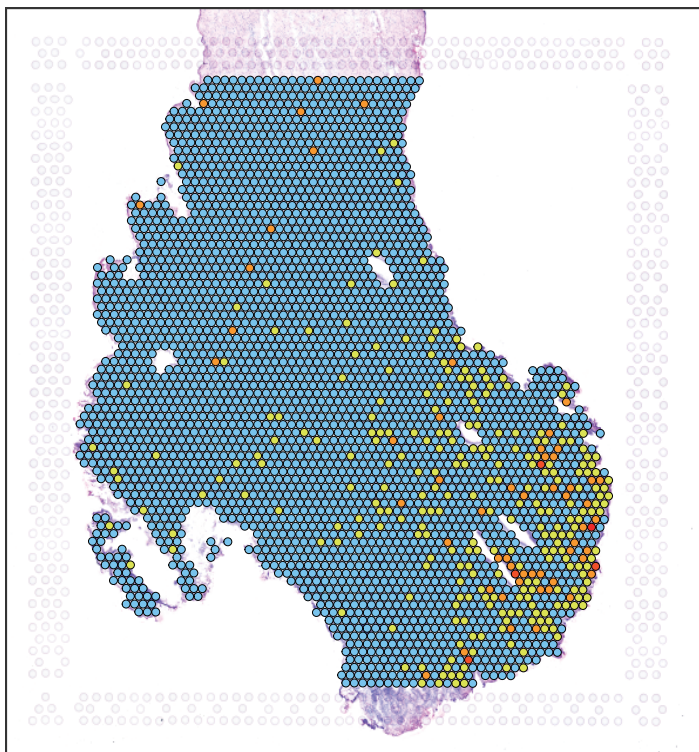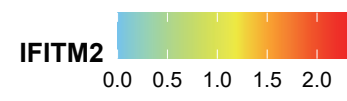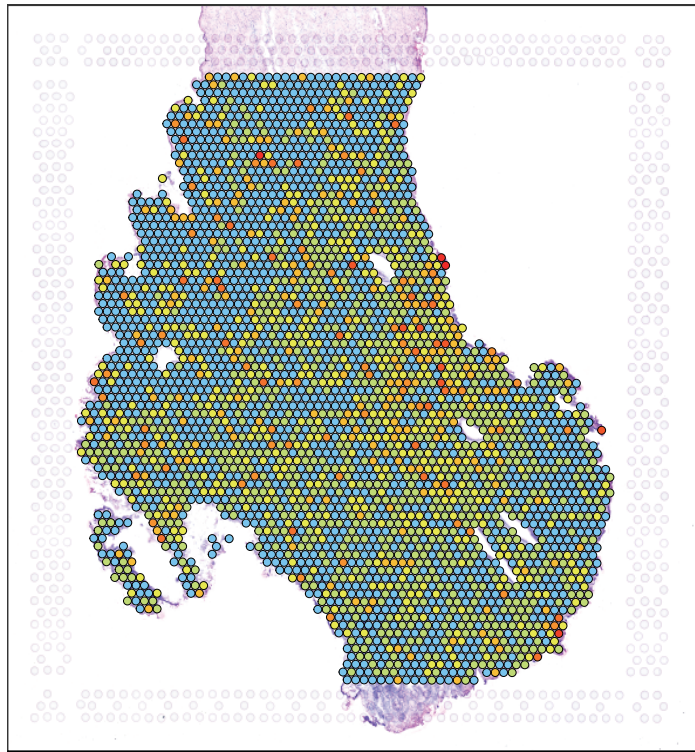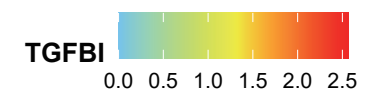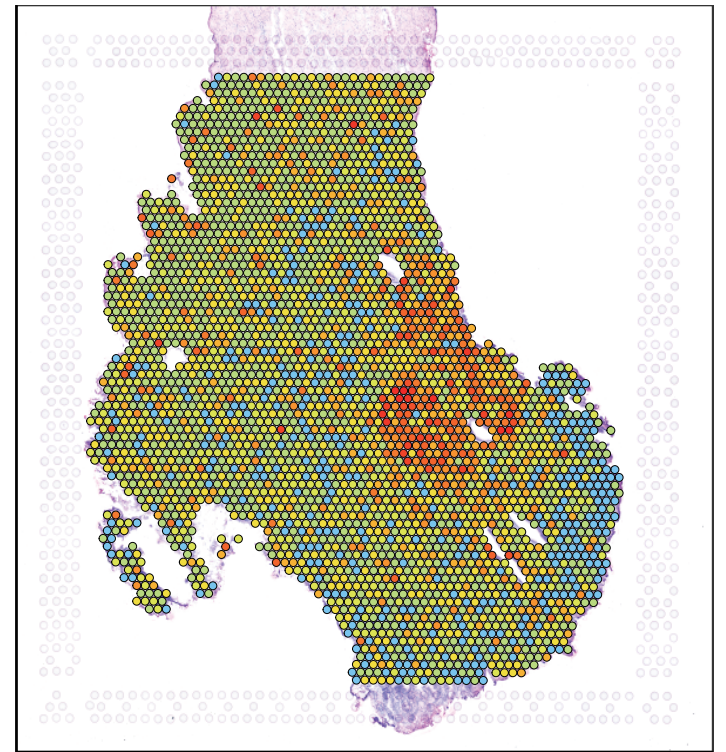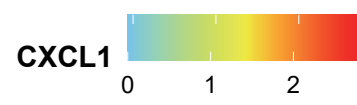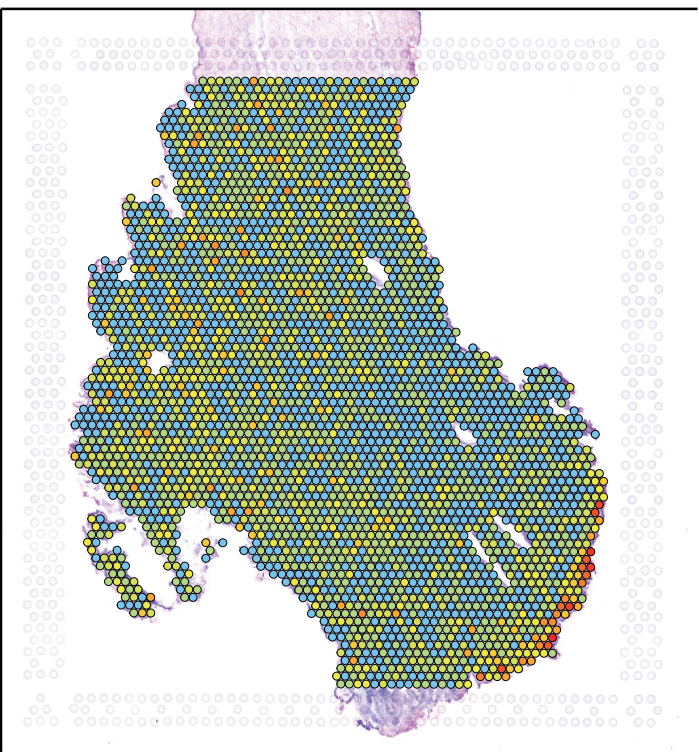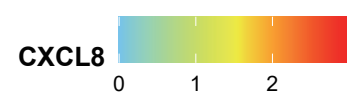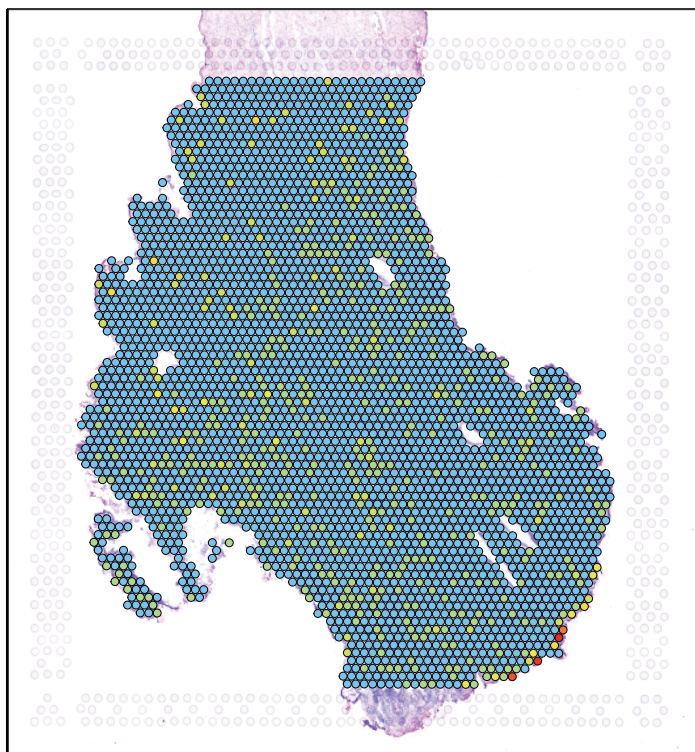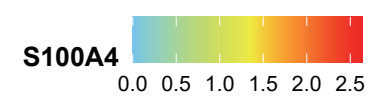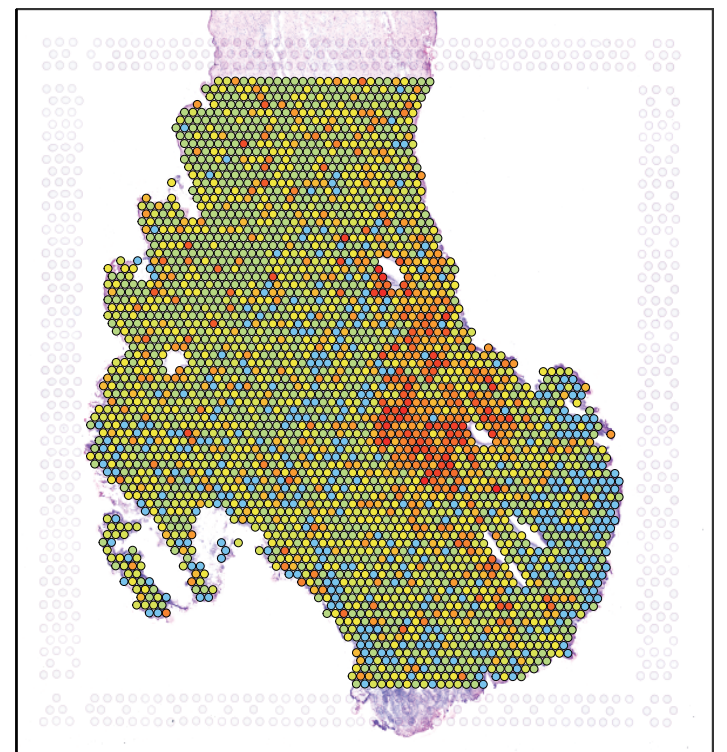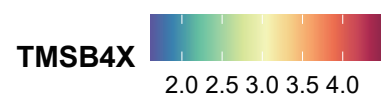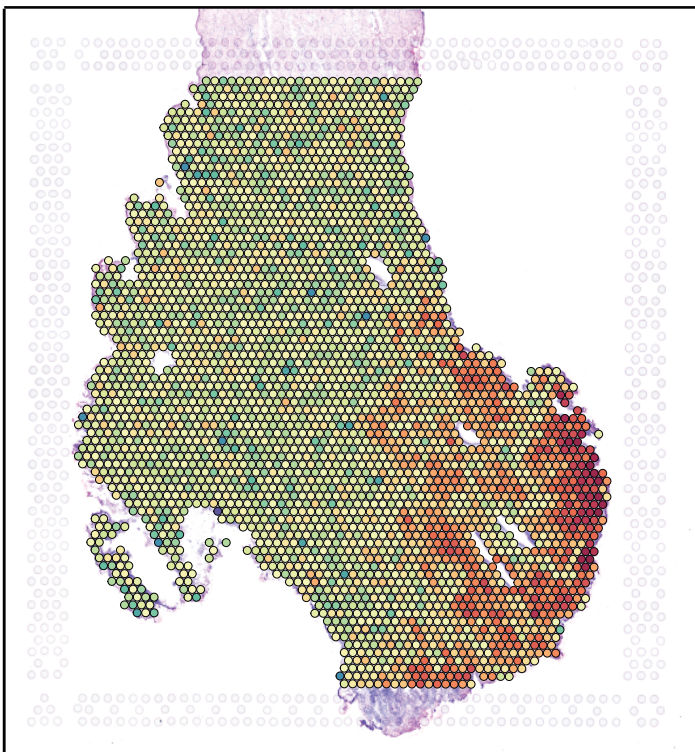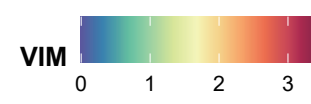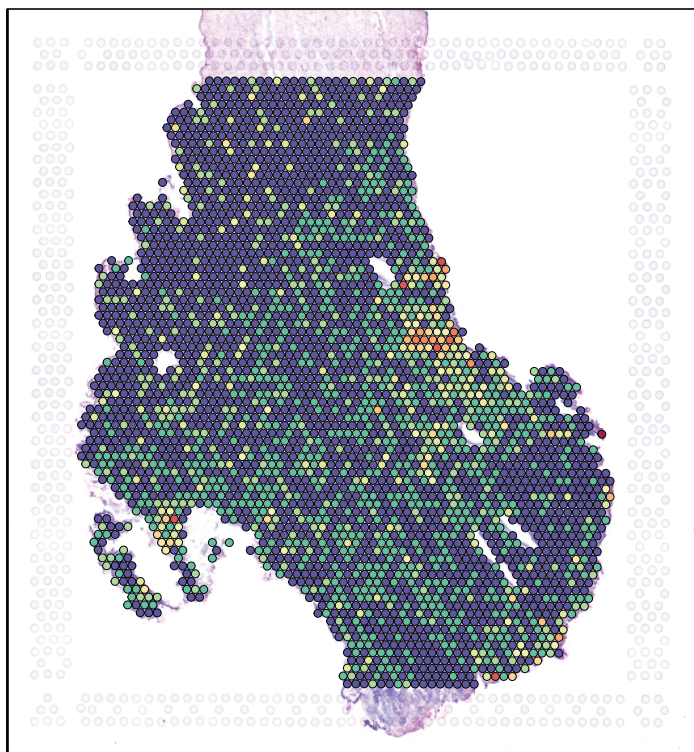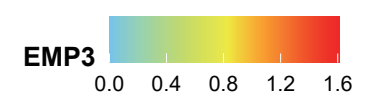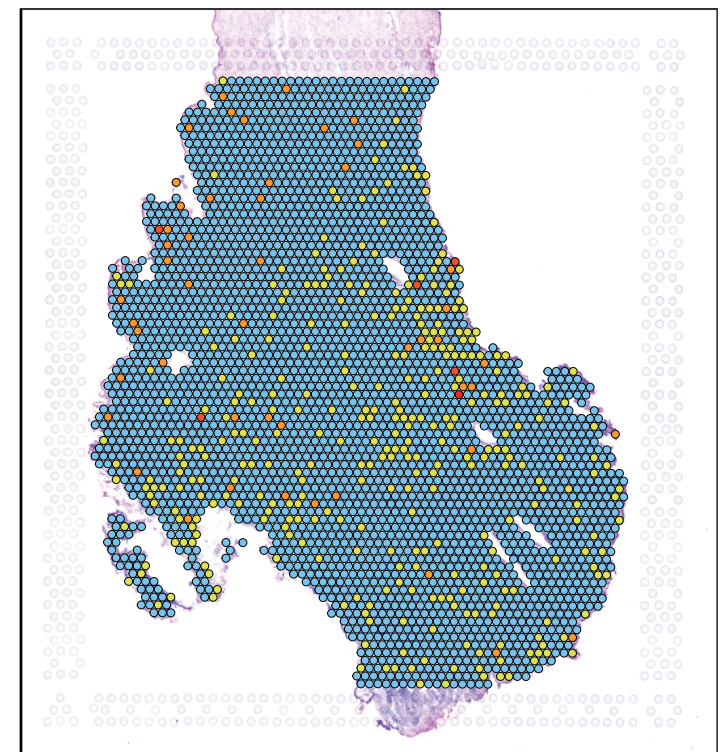

Supplement: Supplementary file 4 — Figure S3. [file 41419_2024_6598_MOESM4_ESM.pdf]

a

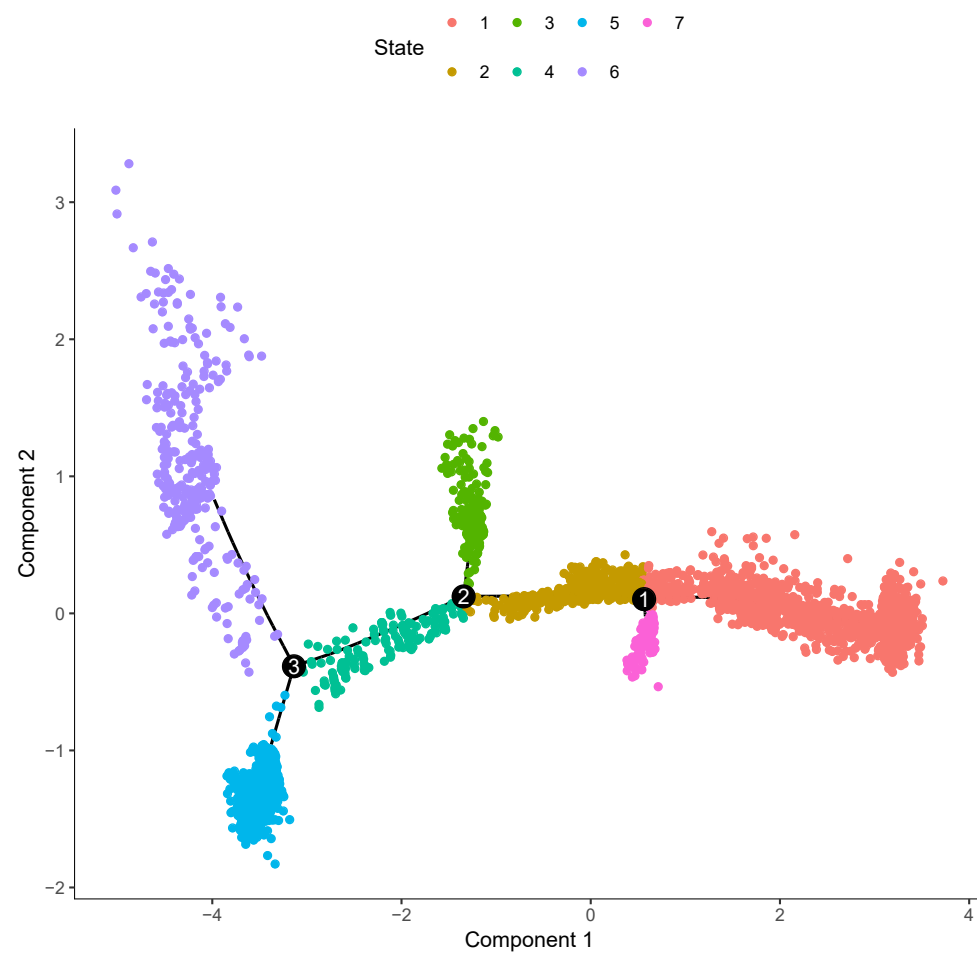

b

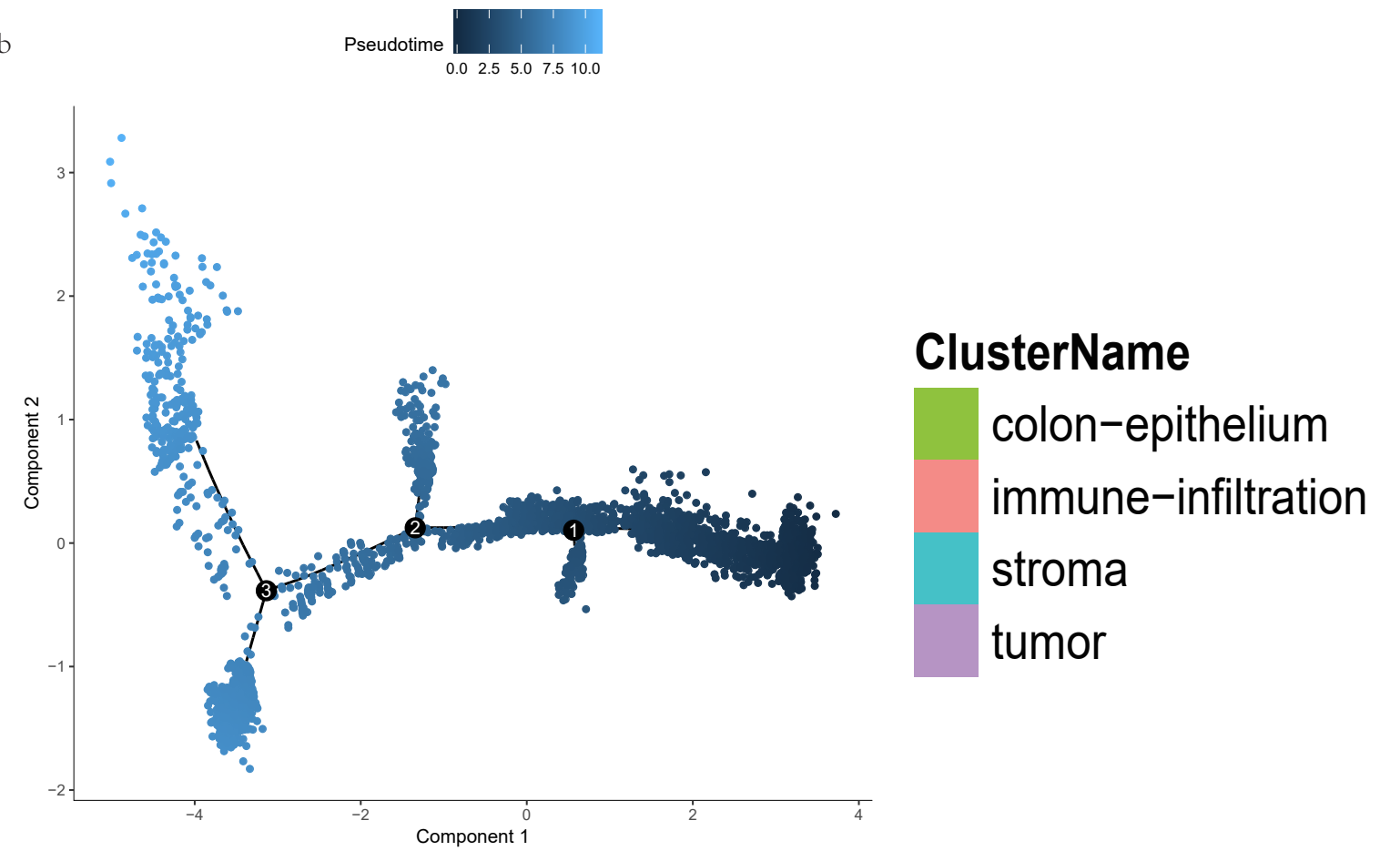

c

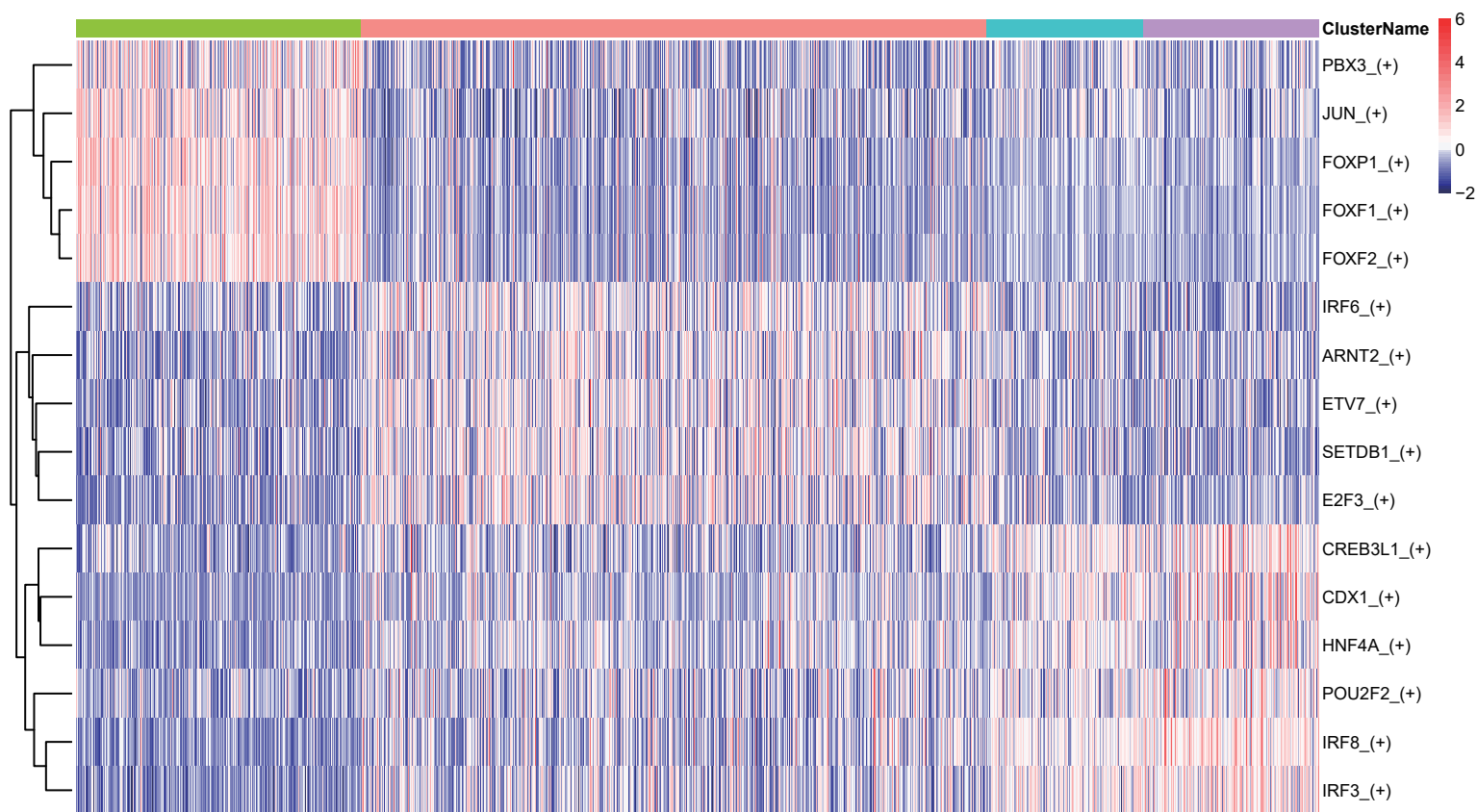

d

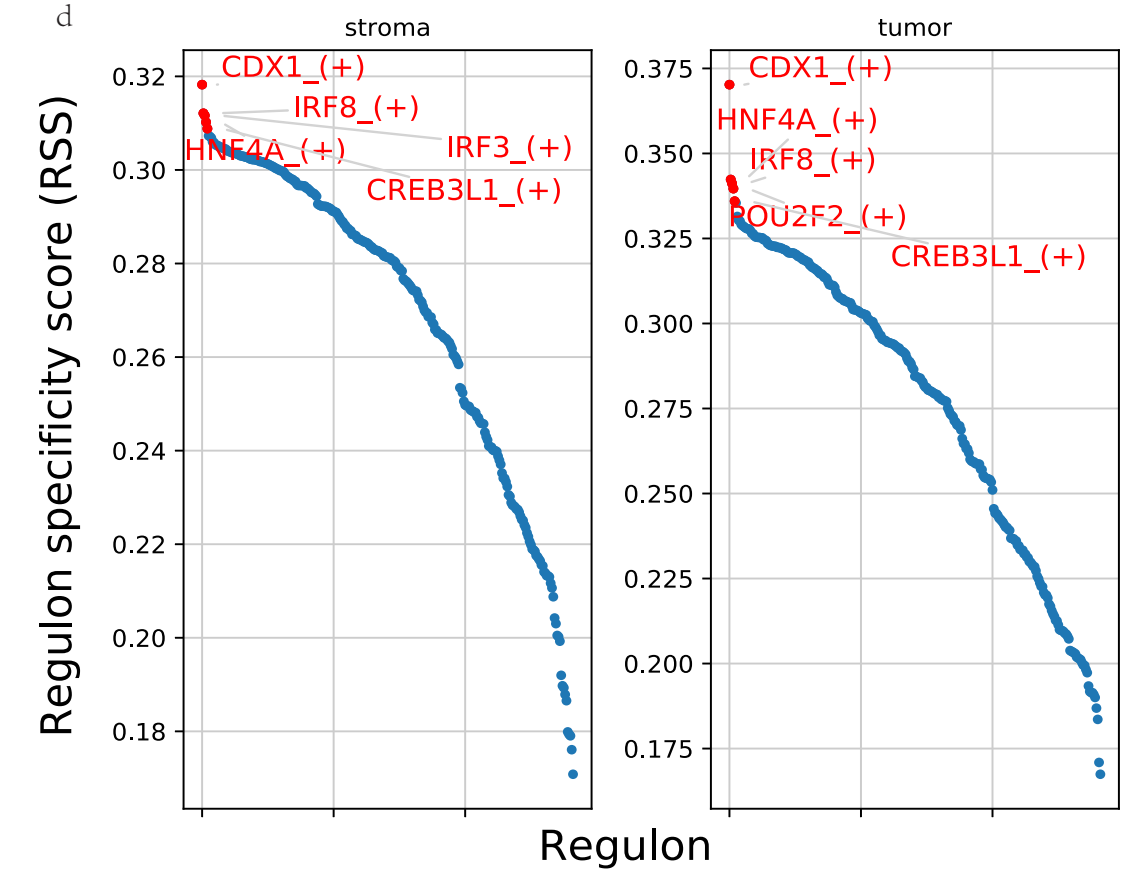

Supplement: Supplementary file 5 — Figure S4. [file 41419_2024_6598_MOESM5_ESM.pdf]
